# Irisin: A Hope in Understanding and Managing Obesity and Metabolic Syndrome

**DOI:** 10.3389/fendo.2019.00524

**Published:** 2019-08-02

**Authors:** Lidia I. Arhire, Laura Mihalache, Mihai Covasa

**Affiliations:** ^1^Department of Internal Medicine, “Grigore T. Popa” University of Medicine and Pharmacy, Iaşi, Romania; ^2^Clinical Hospital “Sf. Spiridon”, Iaşi, Romania; ^3^Department of Basic Medical Sciences, College of Osteopathic Medicine, Western University of Health Sciences, Pomona, CA, United States; ^4^Department of Health and Human Development, University of Suceava, Suceava, Romania

**Keywords:** adipose tissue, thermogenesis, adipokines, exercise, skeletal muscle

## Abstract

White adipose tissue (WAT) is an endocrine organ highly integrated in homeostasis and capable of establishing ways of communicating and influencing multiple metabolic processes. Brown adipose tissue promotes energy expenditure by incorporating the uncoupling protein 1 (UCP1), also known as thermogenin, which decouples cellular respiration and heat production, in the mitochondrial membranes. Recent data suggest the presence of a thermogenic cell formation from white adipocytes (beige or brite cells) with a potential role in preventing obesity and metabolic syndrome. The formation of these cells is influenced by physical exertion that induces expression of PPARγ coactivator-1 (PGC1) and downstream membrane protein, fibronectin type III domain-containing protein 5 (FNDC5) in skeletal muscle. Irisin, a thermogenic adipomyokine produced by FNDC5 cleavage is involved in the browning of adipose tissue. While animal studies are congruent with regard to the relationship between physical exertion and irisin release, the results from human studies are less than clear. Therefore, this review focuses on recent advances in our understanding of muscle and adipose tissue thermogenesis. Further, it describes the molecular mechanisms by which irisin impacts exercise, glucose homeostasis and obesity. Finally, the review discusses current gaps and controversies related to irisin release, its mode of action and its future potential as a therapeutic tool in managing obesity and metabolic syndrome.

## Introduction

Obesity is the pandemic of the Twenty-first century and a worrisome public health issue. Excess weight increases the risk of chronic conditions, such as those referred to as metabolic syndrome and its complications, cardiovascular diseases and stroke, as well as some forms of cancer. Hence the focus on the study of adipose tissue as the main buffer system involved in energy balance. The discovery of the role played by fat cells in metabolic pathologies can render them future therapeutic targets. In addition to “classical” hormones, there are many peptides released by non-endocrine cells that act on the nearby cells (paracrine effects) or on the cells that produce them (autocrine effects). The effects of these peptides are hormone-like, requiring their interaction with the specific cell surface receptors (1). Tissues previously considered “passive” or monospecialized have been shown to be involved in metabolic regulation through endocrine, paracrine and autocrine signaling. Thus, adipose tissue, intestine, skin, and muscle are all dynamic endocrine organs secreting an array of hormones or hormone-like substances with a significant role in maintaining cellular energy homeostasis and regulation of metabolic functions. One such hormone is the recently discovered polypeptide irisin, best known as the exercise-induced chemokine that is released primarily by muscle and adipose tissue, although other tissues including liver, lung, tongue, ovaries, testes, and neuronal cells have been found to express irisin.

Until relatively recently, fat was thought to have a passive role in the development of obesity, with adipocytes being considered storage cells for triglycerides. Adipose tissue has extensive distribution in the body, occupying most of the subcutaneous region, infiltrating organs and tissues, performing mechanical, and thermal protection functions (2, 3). In addition, adipose tissue represents the site of release of numerous adipokines such as leptin, adiponectin, resistin, nesfatin, and irisin, to name a few. These hormones may have local effects based on the fat distribution and type of fat, mediating the link between fat metabolism and overall metabolic and physiologic functions. For example, irisin is released mainly by the white subcutaneous adipose tissue (SAT) and has a key regulatory role in conversion of white fat to brown fat, suggesting its potential role in curbing fat accumulation and obesity and improving metabolic status. From a clinical point of view, namely, in regards to disease progression, two main forms of obesity are recognized: visceral obesity and subcutaneous obesity (4). Visceral adiposity, especially ectopic fat, increases the likelihood of premature death regardless of the body mass index, but in correlation with high abdominal circumference (5). On the other hand, subcutaneous adiposity appears to be benign in terms of the incidence and severity of complications (6). The association between regional fat deposits and the development of obesity was first observed in the 1950s by Vague (7), who also noted that visceral obesity was more common in males while subcutaneous obesity was more prevalent in women (8). Subcutaneous fat transplantation or removal of visceral fat can bring metabolic benefits (9). Animal models have long confirmed the relationship between the site of body fat accumulation and metabolic complications. For example, the overexpression of 11β-hydroxysteroid dehydrogenase type 1 (11-β HSD-1) in the adipose tissue of transgenic mice leads to metabolically unhealthy obesity with insulin resistance (IR) and alteration in glucose and lipid metabolism, while those overexpressing adiponectin or mitoNEET, a key regulator of mitochondrial function and lipid homeostasis, develop subcutaneous obesity and remain metabolically healthy (10, 11). This difference was initially explained by varying degrees of systemic inflammation associated with increased TNFα secretion (12, 13). Similarly, irisin has been associated with reduction of pro-inflammatory cytokines while promoting secretion of anti-inflammatory cytokines in adipose tissue. Given irisin's activity on the target tissues (i.e., muscle and adipose), we will briefly review the mechanisms involved in the adipose and muscle tissue thermogenesis and the role of adipokines and myokines, respectively.

### Adipose Thermogenesis and Adipokines

There are two types of adipose tissue: white or brown. In humans, fat consists mainly of white adipose tissue (WAT), which is highly involved in homeostasis and capable of establishing auto, para-, and endocrine ways of communicating with other tissues and organs. Fat, composed of adipocytes/preadipocytes, also contains endothelial cells, multipotential mesenchymal cells, nerve cells, and immune cells participating in inflammatory and metabolic/hormonal responses. It secretes cytokines called adipokines (adipocytokines) which impact inflammation, angiogenesis, and metabolic processes (14). Some of these adipokines are primarily secreted by the adipocyte (e.g., leptin, adiponectin, resistin, chemerin, and visfatin) but many (e.g., TNFα, IL-6, or MCP-1) are secreted by other cell types as well (15–18). As these molecules can generate signals at local and peripheral level, it is believed that they influence many metabolic pathways as well as the differentiation of adipocytes. They also serve as mediators linking inflammation and immunity with obesity and its comorbidities/complications (19). WAT may in fact be the largest endocrine organ, generating an abundance of hormones, growth and complement factors, and other molecules including receptors for many of these biological agents (20).

It is clear though that WAT mainly stores triglycerides and fatty acids (the largest energy reserve) and is composed of cells with a single (unilocular*)* lipid droplet and few blood vessels, thus resulting its white-yellow appearance. It contains an eccentric nucleus and a very small number of mitochondria. By comparison, brown adipose tissue is specialized in energy expenditure (“burns calories”) (21). It consists of many multilocular lipid droplets, and a very large number of iron-containing mitochondria (21, 22). The mitochondrial membranes of this tissue include a protein called uncoupling protein 1 (UCP1), which pumps protons from the mitochondrial matrix to the mitochondrial intermembrane space (23). Activated UCP1 does not cause ATP synthesis but leads to heat release, regulating body temperature especially in newborns (23, 24). BAT has a positive influence on metabolic processes and increases the total energy expenditure, resulting in body mass reduction (21). BAT produces specific endocrine factors (fibroblast growth factor-21) as well as remote signals with systemic consequences (25). BAT can also increase the uptake of blood glucose and lipids, improving their metabolism independent of weight loss.

**Figure 1:**
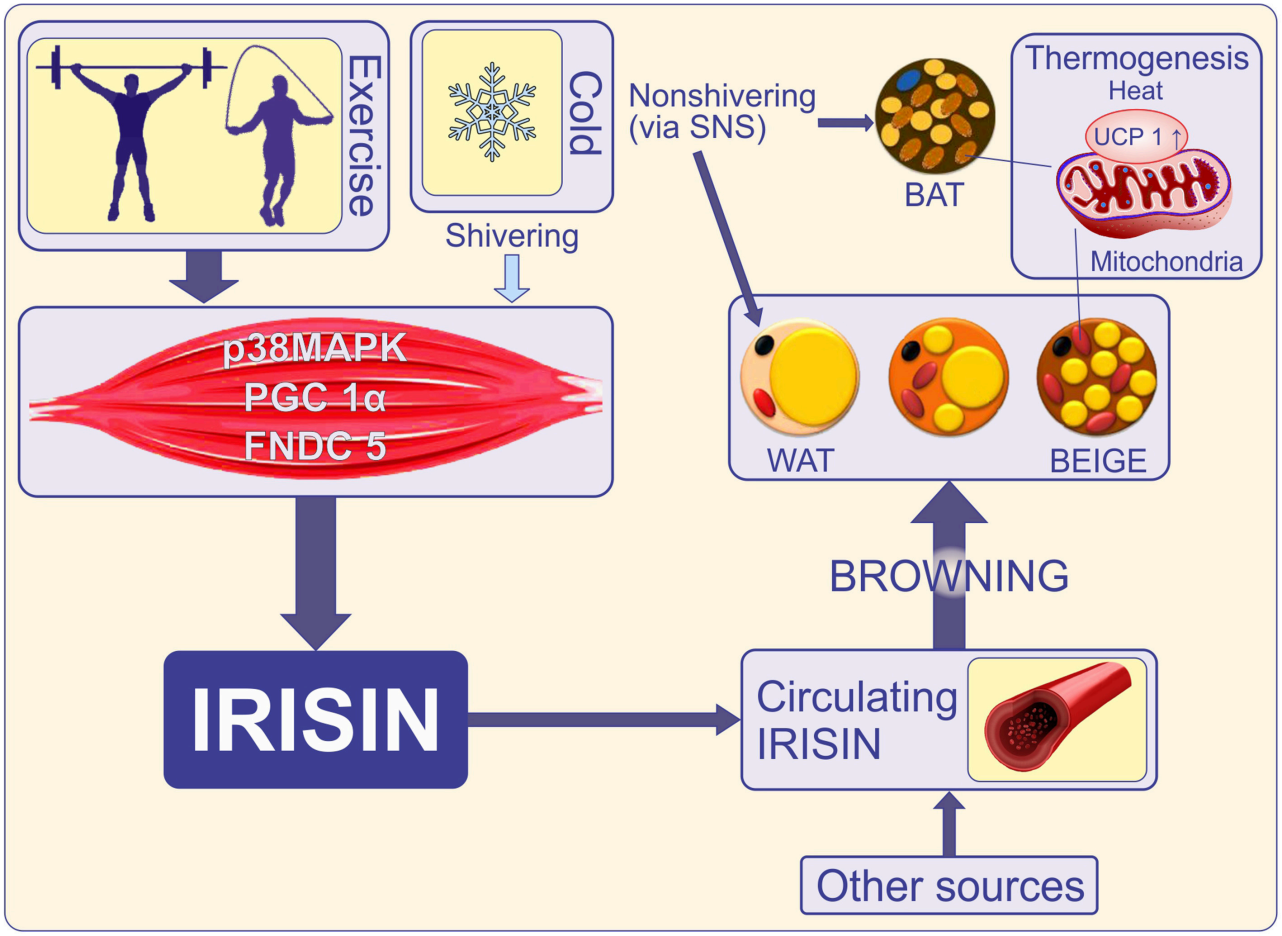
Cold initiates shivering in skeletal muscle and a non-shivering phase in which brown and beige adipose tissue is activated to release heat via UCP-1. Shivering and exercise promote adipose tissue-mediated thermogenesis via secretion of irisin (30). Exercise increases transcriptional co-activator PGC1-α and induces expression of the FNDC5 gene. FNDC5 membrane protein is cleaved to release irisin into the bloodstream. Irisin is also released by adipocytes thus becoming an adipocytokine (18). Other sources such as the brain, pancreas, stomach, Kupffer cells, tongue, rectum, heart, testis, sinusoidal epithelial cells, and optic nerve also release irisin (31). Irisin promotes “browning” of mature white adipocytes in response to exercise (32, 33). Brown and beige adipose tissues increase energy expenditure through the uncoupling of oxidative metabolism from ATP production. This is a key function of UCP1 (34). PGC-1α, Peroxisome proliferator-activated receptor gamma coactivator 1 alpha; FNDC5, Fibronectin type III domain-containing protein 5; WAT, white adipose tissue; BAT, brown adipose tissue; BEIGE, beige adipocyte, brite (“brown-in-white”), or inducible BAT; UCP1, uncoupling protein-1; SNS, sympathetic nervous system.

It is known that BAT is abundant in rodents and low in large mammals, being present in humans during intrauterine life and in infants in the interscapular and perirenal regions, but even these small amounts gradually disappear (26). In adults, BAT is remnant and without significant physiological activity (27). The recent discovery of active BAT in adult humans and the documentation of several transcription factors that regulate the formation of new thermogenic adipocytes makes it attractive to increase this type of adipose tissue and use it as a therapeutic target (28). Subcutaneous fat can be turned “brown” under several stimuli such as cold, beta-adrenergic agonists, or hormone-like stimuli (29) (Figure 1). This fat browning includes UCP1mRNA induction and expression of genes that uncouple respiration and heat production. It has been shown in mice that a decrease in visceral fat is possible, for instance with the genetic ablation of retinaldehyde dehydrogenase 1 (RALDH1) (35), but this process is much less common than WAT browning. The discovery of inducible beige adipocytes has expanded the research in this field and holds therapeutic promise (36).

Animal data provide evidence for the existence of two types of thermogenic UCP1-positive cells: the constitutive BAT or “classical” BAT (cBAT), which appears in the intrauterine life and can be found in the interscapular region of mice and infants, and recruitable BAT (rBAT) located in WAT and muscles, which has been called alternately beige, brite (“brown-in-white”), or inducible BAT. “Classic” cells or “developmentally programmed” brown adipocytes (cBAT) arise from a skeletal muscle lineage (Myf5/Pax7), whereas beige cells (rBAT) originate partly from a vascular smooth muscle–like lineage (as revealed by the Myh11 promoter) (37–41). It seems that a transcriptional regulatory protein called PRDM16 controls this muscle-brown fat decision during the mid-gestation period in mice (39, 42). The postnatal role of PRDM16 in cBAT has not been studied, but it is likely to have a role in the formation and functioning of beige cells (43, 44). It has been demonstrated on several occasions that brown and beige adipocytes are distinct cell types (45), not only in origin (37), but also in their different responses to hormonal stimuli and genetic manipulation (32, 46) and the difference in gene expressions in cell cultures (44). The human brown fat deposits are molecularly more similar to rodent beige fat than to cBAT (34, 44, 47, 48). The factors which selectively induce browning of WAT, like the transgenic expression of PRDM16, showed positive metabolic effects, giving hope that this process may have therapeutic potential (49).

The cBAT and beige adipocytes both express UCP1 and also share other structural and functional characteristics such as the β-adrenergic receptor/cAMP dependent, which regulates the expression of thermogenic genes (50). It is rather difficult to have a complete molecular description of UCP1+ adipocytes *in vivo* because UCP1+ adipocytes in general and beige adipocytes in particular, exist among many other cell types in the adipose tissue. It is also challenging when attempting to separate the functional importance of the browning of WAT from that of cBAT because many browning agents under investigation act on both types of adipocytes. Moreover, it has been suggested in animal studies that it is the UCP1+ cell ablation or the Ucp1 mutation of brown fat cells overall that has a beneficial effect in preventing obesity and diabetes, however the contribution of each type of brown cell to this process remains unclear (51, 52). It is also debatable whether there are sufficient beige cells to generate an effect on a large scale under ambient conditions (53). Nevertheless, it is well-established that physical activity reduces all-cause mortality and increases longevity (54). In particular, exercise decreases the risk of type 2 diabetes mellitus and cardiovascular diseases, has anti-inflammatory effects, reduces visceral adiposity, and stimulates exercise-dependent myokines activity (55).

## Muscle Thermogenesis and Myokines

Muscle is an effector organ, important in thermogenesis, breathing, posture maintenance, locomotion, and generation of power. To meet such metabolically and physiologically demanding roles, muscle uses energy from the stored triglycerides and glycogen. In addition, and in the case of starvation, it calls on its own source and generates lactate and amino acids *via* gluconeogenesis. Over the last decade, the focus has been on the skeletal muscle as an endocrine organ, acting *via* a host of cytokines and other peptides referred to as myokines (56). These myokines have the ability to engage in the cross talk between the muscle and other organs like the adipose tissue, the liver or even the brain. To date, various types of myokines have been identified, such as IL-6, IL-7, IL-15, insulin-like 6, fibroblast growth factor-21, follistatin-like 1, musclin, and irisin. It has been shown that these myokines involve the muscle in various metabolic processes such as myogenesis (57), fat oxidation (58), osteogenesis, endothelial function, and fat browning (32, 59). Myokines can be classified as exercise-induced and independent of muscle contraction, myokines that only influence the muscles (myogenesis, hypertrophy), and its metabolism (60) or myokines that communicate with other remote systems and organs (adipose tissue, bone, pancreas, liver, intestine, brain) (61). Therefore, studying myokines will help answer critical questions leading to our understanding of the mechanisms underlying chronic diseases related to physical inactivity, such as obesity and diabetes.

## Origin of Irisin

Irisin, first discovered in animals and later in humans, is one of the most studied exercise-induced peptides in recent years (32) and has enjoyed increased popularity since its launch with the slogan “light my fire” (62). Irisin belongs to the class of adipomyokines since it acts both in adipose and muscle tissue (adipokine and myokine) and is a thermogenic protein that promotes energy expenditure by WAT browning (63). Irisin dissipates energy as heat (64) and for this reason its discovery has generated a flurry of research aimed at understanding the mechanisms of energy metabolism (65). Its isolation in muscle tissue, nomenclature, and initial chemical characterization was performed by Bostrom et al. who showed that irisin is a protein with 112 amino acids and a molecular weight of 12 kDa (32). The name irisin originates from the ancient Greek goddess Iris (32), daughter of Thaumas and Electra who, in Greek mythology, was the goddess messenger of good news from Gods to humans (66). This name proved to be very appropriate as it relates to the main function of irisin, as a chemical messenger, transmitting the beneficial effects of physical exercise to the adipose tissue (browning and thermogenesis) and other organs involved in metabolism.

## Irisin and Exercise

Bostrom et al. (32) found that physical activity induced in skeletal muscle the transcriptional regulator peroxisome proliferator-activated receptor-γ co-activator 1α (PGC-1α), which is responsible for the synthesis of fibronectin type III domain-containing protein 5 (FNDC5). PGC1α mediates the programming of energy metabolism in transcriptional biological systems, controls mitochondrial biogenesis, angiogenesis, fiber-type switching, and oxidative metabolism in many cell types. FNDC5 is a glycosylated type I membrane protein that is abundantly expressed in skeletal muscles and contains an N-terminal signal peptide and fibronectin type III repeats. FNDC5 (also called FRCP2 and Pep) was discovered and characterized in 2002 at the same time by two independent research groups (67, 68). The C-terminal tail of FNDC5 is in the cytoplasm, whereas the extracellular N-terminal part is released into the circulation as irisin (69–71). Therefore, irisin is a proteolytic cleavage product of FNDC5 in skeletal muscles (32, 69) and is homologous between humans and mice. Consequently, circulating irisin levels are increased in individuals engaged in exercise-induced activities and progressively reduced in those less active and sedentary. Therefore, given the overall beneficial health effects of exercise on cardiovascular, obesity, diabetes, skeletal and other diseases, it is reasonable to believe that irisin may play an essential role in several functions associated with these diseases as well as in other functions as yet to be discovered (18).

Among the several factors that alter the level of FNDC5 and implicitly the level of circulating irisin are cold (30), physical activity (60), and leptin (which increases muscle mass but decreases browning of WAT) (72). As expected, irisin levels correlate positively with the muscle mass (73). As such, in animals, plasma irisin levels increase by 65% after 3 weeks of freewheel running, while in healthy humans irisin levels double after 10 weeks of endurance exercise (32). However, some data did not confirm the activation of the FNDC5 gene by exercise (74, 75), or rather, regular exercise was inversely correlated with irisin levels in adult men (76). These discrepancies have been explained by the fact that irisin levels only increase when more energy is required and this state is observed in the sedentary lifestyle, where ATP concentration is strongly decreased in muscles without physical activity (73). Thus, physical exercise induces expression of PPARγ coactivator-1α (PGC1α) in skeletal muscle and downstream membrane protein, FNDC5, which is proteolytically cleaved forming irisin (Figure 1). Skeletal muscle is a major site of insulin resistance because it uptakes most glucose under the action of insulin. Several experimental studies suggest that irisin influences glucose metabolism in skeletal muscle in an autocrine manner (75). Moreover, the existence of an irisin receptor has been proposed, in the skeletal muscle and it is reasonable to assume that it may be present in other tissue types given that irisin acts at very low concentrations (75). Nevertheless, a model for receptor activation was suggested by Schumacher et al. who reported on the structure of irisin, demonstrating dimerization (77). Further experimental evidence in animal models showed that exogenous FNDC5 induces UCP1 expression in subcutaneous white adipocytes, especially in the inguinal ones, and that FNDC5 overexpression in the liver (indicating increase of systemic irisin) prevented diet-induced weight gain, metabolic disturbances, and stimulation of oxygen consumption (78). Finally, immunohistochemistry of FNDC5-treated adipocytes showed a brown fat-like phenotype with an increase in UCP1 immunoreactivity (32). Taken together, these findings provide accumulating evidence pointing to the mediation of FNDC5/irisin activity in adipose and non-adipose tissue by a receptor-like mediating pathway. Indeed, using *in vitro* cell line cultures and *in vivo* mice models, Kim et al. (79) recently reported that irisin binds to integrins, a class of cell adhesion molecules involved in cellular signaling. Of particular interest are the αvβ5 and α5β1 integrins that bind to arginine–glycine–aspartic acid (RGD) motif of the extracellular fibronectin molecules. Specifically, the authors showed that αv integrin receptors mediate the actions of irisin since its effects on inguinal fat thermogenesis were abrogated by a fibronectin-like RGD molecule. Furthermore, irisin treatment upregulated expression of Ucp1 and Dio2, two main proteins involved in adipose tissue thermogenesis, whereas inhibitors targeting the aV/b5 integrin subset abolished irisin- mediated signaling. These findings corroborate previous work showing that α5 and αv integrins are down-regulated in adipogenesis. In other words, “browning” of fat seems to be the result of irisin-induced attenuation of αv signaling, leading to the suggestion that a certain subgroup of integrin complex acts as irisin receptor via its RDG-homologous motif. It is worth noting that although the integrin complex has been shown to encapsulate the irisin receptors, this finding does not rule out the presence of other irisin receptors within or outside the integrin family. It remains to be seen whether the effects reported in cellular and animal models can be recapitulated in humans and whether FNDC5 protein can also mediate irisin activity and signaling with implications in organ functions.

## Irisin and Glucose Homeostasis

**Figure 2:**
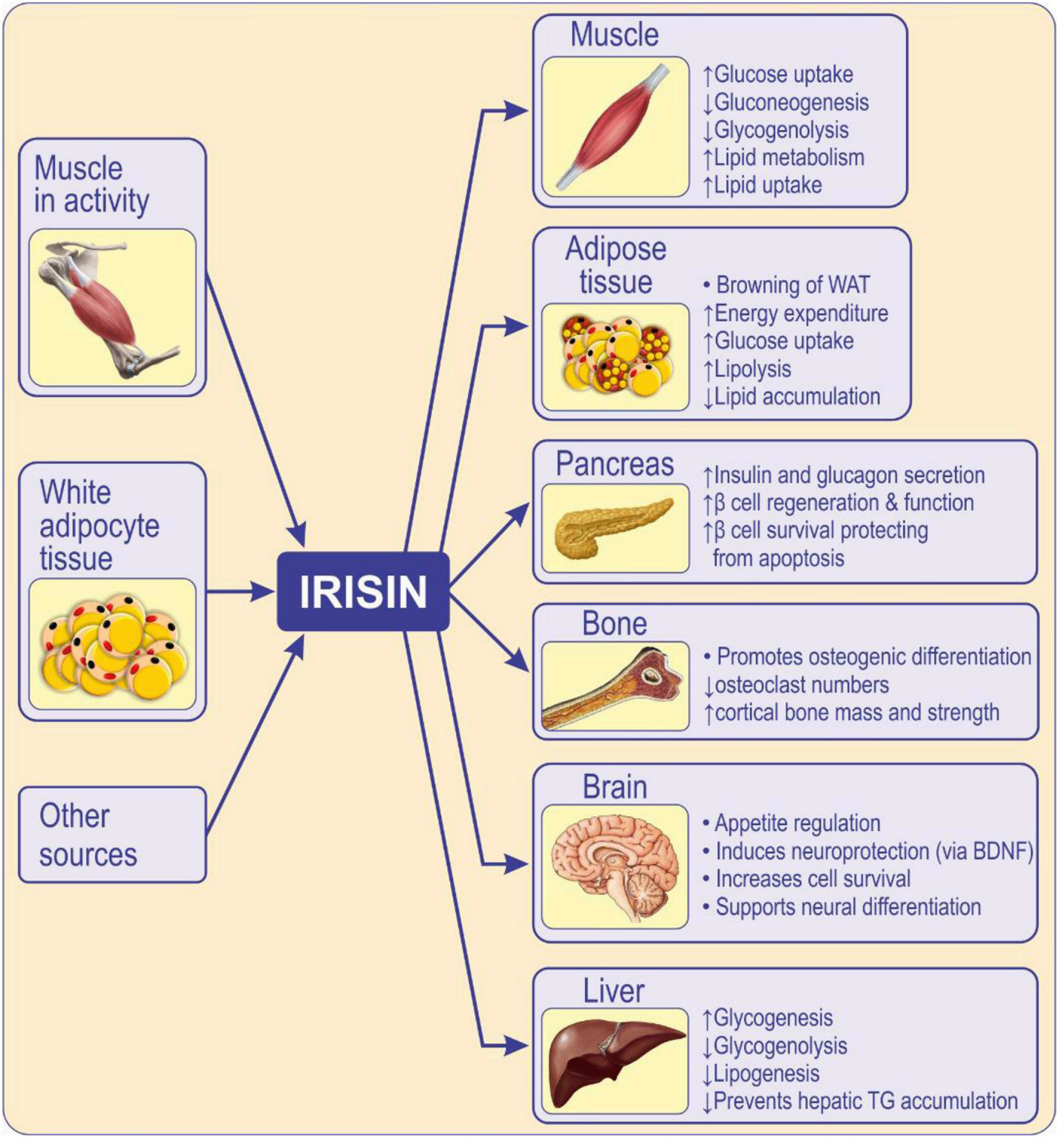
Circulating irisin originates mainly from skeletal muscle during activity of, and from, adipose tissue. Irisin acts locally, in an autocrine/paracrine manner, and when released into circulation, acts in a hormone-like fashion mediating peripheral activity (80). Irisin has multi-spectrum functions on various tissues or organs, inhibits adipogenic differentiation while promotes osteogenic differentiation (59). Irisin influences the function of skeletal muscle, pancreas (81), liver (82), brain (83), and bone (84, 85) enhances insulin sensitivity, metabolism, cognition, and osteogenesis. Irisin improves insulin resistance and type 2 diabetes by increasing sensitivity of the insulin receptor in skeletal muscle and heart, by improving hepatic glucose and lipid metabolism and by promoting pancreatic β cell functions, and browning of white adipose tissue (86). WAT, white adipose tissue; BDNF, brain-derived neurotrophic factor.

Irisin facilitates glucose uptake by skeletal muscles, improves hepatic glucose and lipid metabolism, having a positive effect on hyperlipidemia and hyperglycemia caused by obesity and metabolic syndrome (80), and therefore acts as an insulin sensitizing hormone (Figure 2). It is believed that irisin influences organs and tissues involved in type 2 diabetes, such as the liver and pancreas, by reducing IR, although the mechanisms by which it modulates the function of pancreatic islets are still unknown (60). It is suggested that irisin can improve hepatic metabolism by reducing endoplasmic reticulum stress (ER stress), and that it contributes to β-islet cell mass survival and function (Figure 2). Therefore, irisin may positively impact the liver and pancreatic islets, thus diminishing the risk of developing type 2 diabetes mellitus (80).

The involvement of the liver in glucose homeostasis is to maintain a balance between hepatic glucose production and glucose storage. The rate of gluconeogenesis is primarily dictated by the transcription levels of the gluconeogenesis enzymes, phosphoenolpyruvate carboxykinase (Pepck), and glucose-6-phosphatase (G6Pase). On the other hand, inhibition of GSK-3 promotes activity of glycogen synthase leading to glycogenesis (87). Liver lipid metabolism also plays an essential role in glucose homeostasis. When the intake of carbohydrates exceeds the capacity to store and oxidize, they are converted into fat by *de novo* lipogenesis. However, excess lipids in the liver causes inflammation and IR (88). Considerable evidence has suggested that ER stress, especially hepatic ER, is closely linked to metabolic diseases, by promoting glucose production in the liver, lipogenesis and IR in obesity, and diabetes (89). The activation of hepatic AMPK has anti-diabetic roles in the liver by modulating glucose and fat metabolism, attenuating lipogenesis and gluconeogenesis, and promoting lipid oxidation and glycolysis (90).

Recent studies in animal model have shown that the mechanism by which irisin stimulates glucose uptake in muscle cells involves the calcium/ROS and P38 AMPK mediated AMPK pathway (59, 91). Thus, irisin implicates the AMPK2 activation pathway, probably involving the translocation of p38 MAPK-GLUT4 to the plasma membrane (59, 92). Calcium is also involved in irisin-mediated signaling. These data suggest that irisin could provide a more in-depth molecular explanation for glucose metabolism in muscle with implications in treating diabetes mellitus, as it acts as an insulin-sensitizing hormone (93, 94). Human studies have generated contradictory results regarding the value of circulating irisin in diabetes. For example, lower levels of irisin were found in patients with known diabetes compared to newly diagnosed cases (95) or in subjects without diabetes (96) or values inversely correlated with insulin sensitivity (97, 98). Given the association of irisin with endothelial dysfunction in patients with diabetes, it could be reasonable to expect the use of irisin as a biomarker for the disease or as future therapeutic intervention (99, 100). In some human studies, irisin has been positively associated with risk of metabolic syndrome, cardiometabolic disturbances, and cardiovascular disease, suggesting either an increased secretion in adipocytes or muscle cells and/or irisin resistance in these subjects with an increased compensatory secretion (76). Furthermore, the correlation between irisin and sclerostin levels in female patients with diabetes and atherosclerosis suggests a probable role of irisin in diabetic cardiovascular pathophysiology (100).

## Irisin and Obesity

Although irisin is primarily known as a myokine, it is also released from adipose tissue (18, 60), earning its name as an adipokine and mediating the beneficial metabolic effects of exercise (101). In rodents, FNDC5/irisin is secreted primarily from adipocytes of the SAT and in lower amount from adipocytes of the visceral adipose tissue (VAT) (18). In humans though, FNDC5 expression is 100–200 times lower in WAT than in muscle, indicating a minor contribution of WAT to the circulating irisin levels (102). The contribution of adipose tissue to circulating levels of irisin was confirmed by molecular animal-based studies demonstrating that adipose tissue secretion contributes to circulating FNDC5/irisin (18). Further, adipose tissue of human origin expresses and secretes FNDC5/irisin (103). *In vitro* experiments revealed that irisin is produced in human preadipocytes and adipocytes and in 3T3-L1 adipocytes (73). As a result, irisin precursor PGC1α levels are higher in adipose tissue than muscle following exercise and this correlates with irisin secretion pattern. Moreover, and similar to other adipokines, irisin secretion from SATs is affected by the circulating levels of irisin (18).

Several studies examined the link between circulating irisin, adiposity, and obesity in humans with inconsistent results. For example, some studies reported a positive correlation between serum irisin levels, BMI and adiposity (76, 96, 101, 104, 105), whereas others found a negative correlation between circulating irisin levels, BMI and the amount of fat tissue (101) or could not detect a change in circulating irisin in obesity (71, 73, 75). Similarly, irisin levels in humans were positively correlated with markers of glucose and lipid homeostasis disturbance in obesity and in patients with metabolic syndrome (76, 104). The positive association between irisin and BMI is expected given the increase in muscle mass, however, other tissues, including adipose, may be also involved (60, 73). This is also demonstrated by the positive associations between irisin and fat mass, waist circumference, waist-to-hip ratio and leptin levels (73, 104–106) while a negative association was shown between irisin and adiponectin (107). Furthermore, irisin levels were significantly reduced following weight loss due to bariatric surgery, an effect attributed to a lower fat-free mass and decreased FNDC5 mRNA expression in skeletal muscle (73). On the other hand, the reduction in irisin levels was reversed in patients who regained their original weight (104). This suggests that elevated irisin levels could be a compensatory mechanism for the abnormal metabolism and insulin sensitivity characteristic of obese individuals (73).

Obesity is characterized by a significant imbalance in cytokine secretion that is a strong predictor of developing IR and T2DM (108). In addition to cytokines and lipopolysaccharides (LPS), the activated toll-like receptor 4 (TLR4) is also strongly associated with IR as it increases TNFα expression, that in turn affects insulin signaling pathway in muscle and adipose tissue (109). FNDC5/irisin levels have been associated with glucose metabolism but the likely association between inflammatory markers and irisin levels has not been established. However, it is known that visceral adiposity is associated with high productions of C-reactive protein (CRP) and interleukin-6 (IL-6) and low irisin levels were noted in individuals with high CRP, but not IL-6, levels (106). Further, high FNDC5/irisin levels in middle-aged males with grade 1 obesity were associated with an improved metabolic profile, low risk of developing T2DM, and decreased serum LPS (110). On the other hand, irisin levels were decreased in overweight/obese children with metabolic syndrome, thus, irisin may be used as a biomarker for metabolic syndrome in prepubertal children (111). As such, irisin treatment suppressed expression of pro-inflammatory cytokines, nuclear factor-kappa B (NF-κB), TNF-α, and IL-6 in a concentration dependent manner. Irisin reduced MCP-1 expression in the cultured adipocytes which subsequently attenuated migration of macrophages in the presence of irisin. Moreover, irisin induced the phenotypic switching of adipose tissue macrophages from M1 (pro-inflammatory) to M2 (anti-inflammatory) state (112). Therefore, FNDC5/irisin expression is associated with some anti-inflammatory markers (60). In adipose tissue, irisin stimulates the browning of white adipocytes and thermogenesis by activating the UCP1 (101) whereas blocking irisin gene expression reduces UCP1 expression and enhances adipogenesis in obesity (113). Thus, the inhibitory effects of irisin on adipogenesis highlights its potential role in direct signaling from skeletal muscle to brown adipocytes (101).

Although diet plays a crucial role in regulating metabolic syndrome, no correlations were found between irisin level and diet (76), yet specific diets may be effective in modulating irisin synthesis. For example, serum irisin concentrations increase in patients with metabolic syndrome on a low glycemic index diet (114). Similarly, the supplementation of diet with omega-3 fatty acids (EPA and DHA) significantly increased serum irisin level (115). Omega 3 fatty acids attenuate inflammation and age-associated muscle loss, prevent systemic insulin resistance, and improve plasma lipids. Thus, there is a significant association between circulating irisin and favorable lipid profile in the general population suggesting that increased irisin concentration is associated with low risk for chronic non-communicable diseases (116). This finding is consistent with data showing a positive association of irisin levels with resting energy expenditure seen in obese individuals (105) and a significant reduction following weight loss (73) or in anorexia nervosa (117). Not surprisingly, exercise, and lifestyle changes cause an increase in plasma irisin levels in obesity (107). In fact, irisin levels are negatively associated with waist-to-hip ratio and with waist circumference (106) and a positive relationship between circulating irisin and body mass index has been reported (73, 104, 105, 118). Despite these results, the associations of circulating irisin with body mass, physical activity, exercise training, and dietetic interventions are inconsistent and not completely understood.

## Controversies Over Irisin Secretion and Action

Physical exercise has always been used as an effective tool in the prevention and management of cardiometabolic risk and in the treatment of metabolic syndrome and its complications (119). However, the response to exercise is not uniform, with heterogeneity being influenced by genes and a host of non-modifiable (sex, age) or modifiable (cardiorespiratory fitness, training type, and time) factors. Myokines secreted by skeletal muscle represent one such factor contributing to adaptation to exercise. Among them, irisin is the adipomyokine of great hope for cardiometabolic health, being responsible for the regulation of UCP1 in the formation of beige cells. As noted in the above sections, in has been proved in mice that exercise-induced irisin leads to brown-fat-like thermogenesis in white fat (32). As a result, several studies have searched for uncovering browning formation markers (32, 44, 59, 93) or muscle FNDC5 expression (32, 60, 70, 71) but so far with inconsistent and controversial results. For example, some found that muscle mass is a predictor of irisin level (73) and muscle strength is associated with increased circulating irisin (120). Thus, one would hypothesize that training could lead to acute irisin release. Indeed, this finding was reported by some (32, 70, 121–123), but others did not find such a correlation (124–126). Similarly, some studies reported a positive correlation between irisin release, BMI and other anthropometric parameters (73, 105), while others found a negative correlation (95). It has been postulated that a positive association may represent a compensatory mechanism so that a low irisin level can predict muscle mass loss and onset of sarcopenia (127). The impact of the type of exercise on irisin has been examined in adults with excess weight before and after 8 weeks of aerobic or resistance exercise program. There was a significant improvement in anthropometric parameters and maximum oxygen uptake and muscle strength. In addition, circulating irisin was significantly increased after 8 weeks of resistance training but not after aerobic training suggesting that resistance training could be an effective type of exercise in humans (128). This finding is consistent with other results showing that high-intensity exercise or acute exercise produced a greater increase in irisin levels compared to low-intensity exercise (33). Other studies found no significant correlation between circulating levels of irisin after exercise and the age of the participants, the intensity of the training or the type of exercise; however the level of fitness had a positive impact. Thus, regardless of the initial level of circulating irisin, it seems that a positive fitness level and maintaining a normal BMI can make the effort beneficial (33). Chronic exercise does not seem to influence irisin levels (129). A meta-analysis report that included 12 studies published between 2012 and 2014, of which only three randomized controlled trials, showed that chronic exercise training led to a significant decrease in circulating irisin levels (130). This effect was attributed to possible differences in irisin release as a function of the time of day when exercise is performed (131). For example, studies that tested irisin at various times prior and post exercise raise the hypothesis that irisin levels increase for a limited period of time after exercise, and do not continue to remain elevated.

Irisin is elevated in individuals with IR and features of metabolic syndrome in all age groups: children (132), adolescents (133), or adults (98, 134). This association with IR and cardiovascular risk indicates either increased secretion by adipose/muscle tissue and/or a compensatory increase in irisin to overcome its resistance (76). Thus, individuals with elevated circulating irisin levels are more prone to develop IR shortly after gaining weight following a weight loss diet plan (104). On the other hand, energy restriction may be detrimental in obese who develop IR, an effect that could lead to T2DM. As such, serum irisin is increased in non-diabetic obese individuals and decreased in the diabetics (135) with no significant difference between non-diabetics and newly diagosed ones of the same age and sex. Furthermore, in the non-diabetic subjects, irisin is positively correlated with HOMA-β suggesting that it may influence pancreatic β-cell function (136). These variations and inconsistencies between results may be due to a host of factors including but not limited to, physiological and experimental approaches, animal *vs*. human studies, sex, age, health status, BMI, antropometric parameters and body composition, whether the subjects were previously trained or sedentary, the type and duration of training, the timing of irisin determination, the cardiometabolic risks, etc. Furthermore, a wide variety of assays have been used for irisin determination employing various methodologies on either fresh or frozen samples (31, 137). Therefore, further work is necessary to include control studies in order to confirm not only the role of irisin in metabolic diseases but to elucidate its mechanisms of action. Notwithstanding the current drawbacks, irisin remains a hope in prevention and treatment of metabolic syndrome, an effect that needs to be validated in future work.

## Author Contributions

LA, LM, and MC participated in drafting, editing, and writing the manuscript, approved the final version of the manuscript. LA designed the figures.

### Conflict of Interest Statement

The authors declare that the research was conducted in the absence of any commercial or financial relationships that could be construed as a potential conflict of interest.
